# Social-coalitional trait is related to coping capacity with mortality threat: association with leadership and a reduced parietal response to mortality salience

**DOI:** 10.3389/fnbeh.2023.1188878

**Published:** 2023-07-13

**Authors:** Kanan Hirano, Kentaro Oba, Toshiki Saito, Ryuta Kawashima, Motoaki Sugiura

**Affiliations:** ^1^Institute of Development, Aging and Cancer, Tohoku University, Sendai, Japan; ^2^International Research Institute of Disaster Science, Tohoku University, Sendai, Japan; ^3^Faculty of Science and Engineering, Waseda University, Tokyo, Japan; ^4^Japan Society for the Promotion of Science, Tokyo, Japan

**Keywords:** death threat, mortality salience, terror management, fMRI, attachment style, leadership, disaster, parietal cortex

## Abstract

**Introduction:**

Coping with mortality threat, a psychological threat unique to humans and distinct from general emotional distress, is traditionally characterized by immediate suppression and prolonged worldview defense within the framework of the influential terror management theory (TMT). Views regarding the personality-trait concepts for this coping capacity diverge: some favor a broad definition based on general psychological attitudes (e.g., hardiness), while others prefer a narrow definition linked to interpersonal attitudes related to social coalition (e.g., attachment style and self-transcendence).

**Methods:**

Using functional MRI, we presented healthy older participants with death-related words and explored correlations between the neural responses to mortality threat and the factor scores of the Power to Live questionnaire, which measures eight resilience-related psychobehavioral traits.

**Results:**

We observed a significant association between the factor score and a neural response only for leadership; individuals with a high leadership score exhibited reduced neural response to mortality salience in the right inferior parietal lobule.

**Discussion:**

Within the TMT framework, our findings align with the concept of the immediate suppression of death-thought accessibility associated with a secure attachment style, a trait conceptually linked to leadership. These findings highlight the unique role for the narrowly defined social-coalitional trait during the immediate stage of the coping process with mortality salience, in contrast to the broadly defined resilience-related personality traits associated with a prolonged worldview defense process. The deterioration of this coping process could constitute a distinct aspect of psychopathology, separate from dysfunction in general emotion regulation.

## 1. Introduction

Mortality salience, or the awareness that one’s death is inevitable, poses a unique psychological threat to humans. According to the influential terror management theory (TMT), the coping response to this threat enhances state self-esteem or leads to interesting human behaviors, including punishment of cultural norm violators, nationalistic acts, and racial prejudice, namely, worldview defense (Burke et al., 2010). This process is assumed to be distinct from general emotional distress coping mechanisms, despite ongoing debate concerning the process-specificity of the threat-induction methods (Lambert et al., 2014) and its connection to mental disorders (Simon et al., 1998; Menzies et al., 2021). TMT assumes two processes by which individual differences in the coping response are exhibited: first, when a reminder of death is being presented, the degree of increase in accessibility to death-related thoughts varies across individuals (Hayes et al., 2010). Second, the worldview defense involves a prolonged unconscious process that varies across individuals (Burke et al., 2010).

Researchers have proposed that several personality traits related to psychological resilience are also associated with coping capacity for mortality threats, but they vary in terms of the level of conceptual breadth. A broadly defined resilience trait is that of hardiness, a constellation of personality characteristics that function as a resistance resource in encounters with stressful life events (Kobasa et al., 1982). This trait describes psychological attitudes known as the “3 Cs,” commitment, control, and challenge, which are common across multiple resilient-personality characteristics. Hardiness decreases the effects of stressful life events (e.g., a military setting) with regard to producing illness (Maddi, 2007). In the context of TMT, under an experimentally induced mortality threat, hardiness was associated with less worldview defense, without an effect on accessibility to death-related thoughts (Florian et al., 2001). Some investigators have discussed the resistant effect of this trait against mortality threat within the framework of self-esteem, which is also a broadly conceptualized psychological resource that buffers existential anxiety (Joireman and Duell, 2007).

Two other views are related to more specific or narrowly defined resilience-related personality traits in the domain of interpersonal attitudes related to social coalition. One view focuses on the secure attachment style as a key coping ability for mortality threat. This style is based on the belief that proximate others are supportive at one’s time of need or distress (Bowlby, 1973; Waters and Waters, 2006) and provides psychological resilience and adjustment (Mikulincer and Shaver, 2016). In the context of TMT, after a reminder of death, secure attachment is associated with a lesser degree of death-thought accessibility and worldview defense compared to avoidant or anxious-ambivalent attachment (Mikulincer and Florian, 2000; Taubman-Ben-Ari et al., 2002).

Another view considers the self-transcendent personality trait as an important coping ability for mortality threat. Self-transcendence refers to expansion beyond the boundaries of the self in diverse dimensions, including physical and social; it often also includes expanded, prosocial, spiritual, and religious worldviews (Garcia-Romeu, 2010). In the context of TMT, a study demonstrated that worldview defense, in terms of an increase in the importance of charity activity (for people or the environment), after mortality salience manipulation was lower in people with a higher self-transcendence value (benevolence and universalism) orientation (Joireman and Duell, 2007). Another study showed that Christians were less vulnerable than atheists to a reduction in the meaning of life when reminded of death (Vail and Soenke, 2018).

Neuroimaging studies have not examined the associations of resilient-personality traits with the processing of mortality threat. Multiple studies have examined the neural response to mortality threat, typically contrasting it with negative emotional conditions (e.g., dental pain). They demonstrated increased activity in the amygdala (Quirin et al., 2012) and various frontoparietal regions (Yanagisawa et al., 2016; Klackl et al., 2018; Qin et al., 2018; Hirano et al., 2021), decreased activity in the insular cortex (Han et al., 2010; Shi and Han, 2013; Klackl et al., 2014; Qin et al., 2018), and increase (Quirin et al., 2012) and decrease (Qin et al., 2018) in the activity in the anterior cingulate cortex. However, few studies have investigated individual differences in neural activity. Three studies have exhibited conflicting effects of self-esteem (Klackl et al., 2014; Yanagisawa et al., 2016; Luo et al., 2017). Other studies have demonstrated the effects of serotonin transporter gene polymorphisms, interdependence (Luo et al., 2017), sex (Qin et al., 2018), and fear of death (Hirano et al., 2021), whereas no study has examined the associations with resilience-related personality traits.

The recently developed Power to Live questionnaire (Sugiura et al., 2015) can be used to evaluate the different views regarding the resilient-personality trait to mortality threat, owing to its multidimensional structure that encompasses different levels of personality traits. The Power to Live questionnaire measures eight major psychobehavioral characteristics related to survival, which were identified by factor analysis based on interviews of 1,400 survivors of the 2011 Great East Japan Earthquake and Tsunami disaster. The eight factors, namely, leadership, problem solving, altruism, stubbornness, etiquette, emotion regulation, self-transcendence, and active wellbeing, comprise a constellation of resilient personality characteristics related to commitment, control, and challenge; thus, they are relevant to hardiness. On the other hand, specific Power to Live factors, leadership and self-transcendence, correspond to narrowly defined resilient-personality trait concepts related to social coalition, i.e., secure attachment style and self-transcendence, respectively. Leadership describes the social-coalitional response to distress, similar to the secure attachment style, and is composed of items such as, “To resolve problems, I gather together everyone involved to discuss the matter,” and, “In everyday life, I often take the initiative to gather people.” These items appear to reflect a sense of secure attachment, i.e., an implicit belief that proximate others are supportive at one’s time of need or distress (Bowlby, 1973; Waters and Waters, 2006). This factor has been shown to provide psychological resilience and adjustment during tsunami evacuation (Sugiura et al., 2019, 2020), emergency problem-solving (Sugiura et al., 2021), and housing recovery (Sato et al., 2021) in the disaster aftermath. Self-transcendence is evaluated using items such as, “I am aware that I am alive, and I have a sense of responsibility for my life,” and, “I am aware of the path and teachings I should follow as a person.” This is in line with the conventional concept of self-transcendence and overlaps with religiosity, which is related to the ability to cope with a mortality threat (Joireman and Duell, 2007; Vail and Soenke, 2018). This factor has been behaviorally demonstrated to be associated with the sense of self-agency (Niikuni et al., 2022) and to have an adaptive and prosocial nature (Sugiura et al., 2020; Sugiura, 2022). Furthermore, emotion regulation appears to reflect the capacity to cope with general emotional distress (Sugiura et al., 2023) rather than with mortality threat.

In the present functional magnetic resonance imaging (fMRI) study, we examined whether views regarding resilience to mortality threat are supported by neural evidence. We presented healthy older participants with death-related and death-unrelated negative (i.e., control) words, and evaluated correlations between differential neural activation and the eight factors of the Power to Live questionnaire. We were initially interested in the breadth of factors showing a correlation. The view that the broadly defined resilient-personality trait, hardiness, provides coping capacity for the mortality threat predicts that multiple Power to Live factors will be associated with the neural response to mortality salience, probably by reducing the neural response to mortality threat (e.g., reduced activation of the amygdala and frontoparietal cortices and increase in the activation of insula). On the other hand, the view that the narrowly defined resilience-related personality traits in the domain of social coalition provide coping ability seemed to predict the association with a specific Power to Live factor, namely, leadership or self-transcendence. We anticipated that there would not be an association with the emotion regulation factor, given its link with coping capacity for general emotional distress.

## 2. Materials and methods

### 2.1. Dataset

This study analyzed a dataset that had originally been obtained for different research purposes (Hirano et al., 2021). In the original experiment, participants were asked to judge the relevance of the presented word to themselves or other individuals. The prior study identified the self-specific neural response to the death-related word and its association with the fear of death. For the current study, we focused on the correlations between the Power to Live factors and the neural responses to death-related words, without distinguishing between the judgment type (i.e., self vs. other). The study protocol was approved by the Institutional Review Board of the Graduate School of Medicine of Tohoku University, Japan, and was conducted in accordance with the Declaration of Helsinki.

### 2.2. Participants

The dataset was obtained from 34 community dwelling older individuals (aged 66.3 ± 3.9 years; range: 60–72 years; 19 males and 15 females). All participants were right-handed native Japanese speakers, had a high school-level or higher education, were not using medications for hypertension or diabetes, and had no past or present neurological or psychiatric illnesses. Written informed consent was obtained from all participants. See Hirano et al. (2021) for further details.

### 2.3. Questionnaires

The Power to Live questionnaire (Sugiura et al., 2015) includes 34 items related to thinking style, attitude in daily life, and habits; some of these items are related to stressful situations. While the original questionnaire was developed from a survey of disaster survivors, the factor structure was validated in normal adults (Ishibashi et al., 2019) and younger (Matsuzaki et al., 2022) populations. The participants rated the self-applicability of each item on a 6-point scale (0: not at all; 5: very much). Each factor consisted of three to five items; the summed item scores were used to calculate the factor score based on the percentage of the maximum value (% maximum). The representative items are presented in Table 1.

**TABLE 1:** Responses to the Power to Live questionnaire.

Factor	Representative item	Mean score (SD)	Cronbach’s α	Correlation with self-esteem (*r*)
Leadership	To resolve problems, I gather together everyone involved to discuss the matter.	61.4 (17.4)	0.878	0.562**
Problem solving	When I am fretting about what I should do, I compare several alternative actions.	73.1 (11.5)	0.681	0.475**
Altruism	I like it when other people rely on me and are grateful to me.	66.1 (18.1)	0.851	0.122
Stubbornness	I am stubborn and always get my own way.	56.7 (16.3)	0.767	−0.184
Etiquette	On a daily basis, I take the initiative in greeting family members and people living in the neighborhood.	87.3 (12.0)	0.751	0.085
Emotion regulation	During difficult times, I endeavor not to brood.	72.2 (11.9)	0.718	0.302
Self-transcendence	I am aware that I am alive, and I have a sense of responsibility for my life.	73.5 (12.9)	0.701	0.330
Active wellbeing	In everyday life, I have habitual practices that are essential for relieving stress or giving me a change of pace.	74.5 (14.6)	0.673	0.358*

A representative questionnaire item, mean and standard deviation (SD) of the factor score (% maximum), Cronbach’s α, and Pearson’s correlation coefficient (r) with the self-esteem score are shown for the Power to Live questionnaire factors. *p < 0.05, uncorrected; **p < 0.05/8 (Bonferroni correction: threshold p is divided by the number of tests).

The dataset also included the self-esteem scores from the Rosenberg Self-Esteem scale (Yamamoto, 1982; Rosenberg, 2015). Considering the relevance of this trait to TMT (Burke et al., 2010), and to allow comparison with previous neuroimaging studies (Klackl et al., 2014; Yanagisawa et al., 2016; Luo et al., 2017), associations of the self-esteem score with the Power to Live questionnaire scores (Table 1) and neural activity were analyzed.

### 2.4. Stimuli

The participants were presented with 40 death-related words (e.g., sudden death, cremation, and metastasis) and 40 death-unrelated negative words (e.g., stomachache, cold, and eye disease). Each word was a noun consisting of two Japanese Kanji (Chinese characters). The former set had higher death relatedness than the latter. The arousal, emotional valence, imageability, familiarity, and self-relevance were matched between the two word sets in a preparatory behavioral experiment (see Hirano et al., 2021 for details).

### 2.5. Task and procedure

The task comprised a factorial design composed of two stimulus types [death-related (D) and death-unrelated (ND) words] and two task types [judgments related to self (S) and another (O; prime minister)]. As a result, there were four task conditions: DS, DO, NDS, and NDO. Each participant alternated between the S and O blocks. Each block consisted of five trials composed of pseudorandomly ordered two or three D and ND words. Each word was displayed for 6 s, during which a self-relevance judgment (“How relevant the word is to you”) or an other-relevance judgment (“How relevant the word is to the prime minister”) was required using a four-point scale (“relevant” to “irrelevant”) by pressing one of the four response keys. The inter-stimulus interval was 1–9 s, during which a fixation cross was presented. The experiment included a total of four runs (160 trials in total), each containing eight blocks (four S and four O blocks). Each task block lasted for 60 s and began with a 5-s instruction screen that indicated the task type. Each stimulus was presented twice during the experiment (i.e., once in the S block and again in the O block). The visual stimulus was projected on a semi-lucent screen attached to the head-coil of the MRI scanner and was viewed via a mirror (see Hirano et al., 2021 for further details).

### 2.6. MRI data acquisition

For functional data, 38 transaxial images (echo time = 30 ms, flip angle = 81°, slice thickness = 3.0 mm, slice gap = 0.5 mm, field of view = 192 mm, matrix = 64 × 64, and voxel size = 3 × 3 × 3 mm) covering the entire cerebrum were acquired with a repetition time of 2,500 ms using a gradient-echo echoplanar imaging sequence and a 3-Tesla Philips Achieva scanner (Philips Medical Systems, Best, the Netherlands). In each run, 197 volumes were acquired during a total scanning period of 492.5 s; in total, 788 volumes were acquired in four runs. After the runs, T1-weighted anatomical images (thickness = 1 mm, field of view = 192 mm, and matrix = 240 × 240) were acquired using a magnetization-prepared rapid gradient-echo pulse sequence.

### 2.7. MRI data analysis

Statistical Parametric Mapping software (SPM12; Wellcome Department of Imaging Neuroscience, London, UK) was used for preprocessing and statistical analyses. The following procedures were performed for preprocessing using the default parameter settings of the software: correction for head motion, adjustment of acquisition timing across slices, co-registration of the anatomical image to realigned functional images, segmentation of the structural image into six tissue classes, spatial normalization to the Montreal Neurological Institute template using the co-registered anatomical image, and smoothing with an isotropic Gaussian kernel with an 8-mm full-width at half-maximum.

A conventional two-level approach for a multi-subject dataset was adopted for statistical analyses of the fMRI data. In the first-level analysis, the degree of neural activation was estimated based on a voxel-by-voxel multiple regression analysis of the time-series models of neural blood oxygenation level-dependent signals. For each participant, a general linear model was developed using the hemodynamic response function. The general linear model contained regressors that represented the four conditions (i.e., DS, DO, NDS, and NDO). Each regressor modeled neural activation during the 6-s period in which the stimulus was presented. Six estimated head-motion parameters (three for translation and three for rotation) were also included as covariates of no interest. A high-pass filter with a cutoff frequency of 1/128 Hz was applied for detrending. A contrast image D–N [i.e., (DS + DO)–(NDS + NDO)] was generated for each participant to identify the neural response to mortality salience.

In the second-level analysis, to identify the cortical areas in which the neural response to mortality salience was correlated with the scores of the Power to Live factors or self-esteem, voxel-wise multiple regression analyses were performed. Age and sex were included as covariates of no interest. The activation clusters of significant effects (i.e., regression coefficients) were identified, based initially on a voxel-level threshold of *p* < 0.001 (uncorrected) and finally on a cluster-level threshold (*p* < 0.05). The cluster-level threshold is used to correct for multiple comparisons in terms of searching activation over the entire brain applying a voxel-size threshold based on the random field theory (Friston et al., 1994). As we performed this analysis for each of the nine trait scores (i.e., eight factors of the Power to Live and self-esteem) for both positive and negative effects, we also reported the results after correcting for multiple comparisons in this term (i.e., testing multiple traits) using the Bonferroni method (i.e., dividing the *p*-value threshold by the number of tests; 0.05/18 = 0.0028).

## 3. Results

### 3.1. Behavioral data

Table 1 presents the basic statistics, Cronbach’s α, and correlations of the self-esteem score with the eight factors of the Power to Live questionnaire. All factors had good internal reliability (i.e., Cronbach’s α > 0.6). The self-esteem score had significant positive correlations with leadership and problem-solving factors.

### 3.2. fMRI data

**FIGURE 1:**
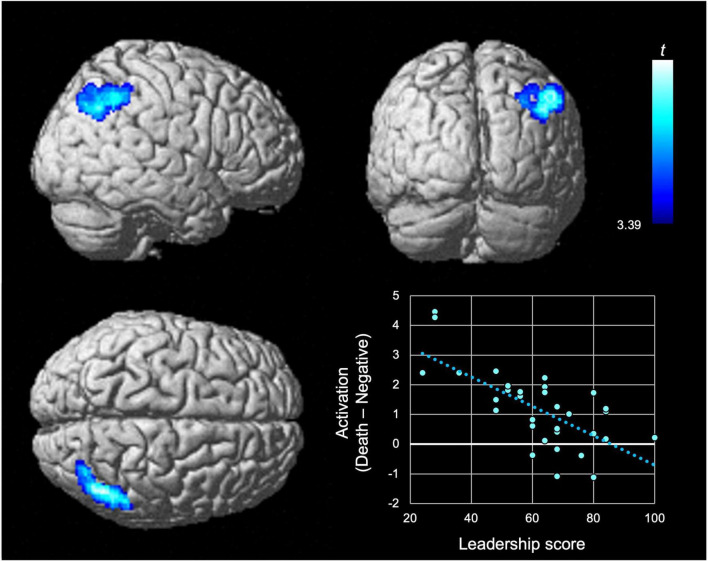
Negative effect of the leadership score on the neural response to mortality salience. Significant effects (*t*-values) of the leadership score from the Power to Live questionnaire in the second-level multiple regression model are represented by a blue-cyan scale, rendered on the surface of an SPM12 standard structural brain image (from the right, back, and top of the brain in the top left, top right, and bottom left panels, respectively). Statistical significance was determined using a cluster-forming threshold of uncorrected *p* < 0.001, further corrected to *p* < 0.05 (family-wise error) to account for multiple comparisons across the entire brain using cluster size, as well as testing the positive and negative effects of nine trials. No positive trait effects were detected. The bottom right panel shows the scatter plot of differential activation (i.e., Death–Negative) against the leadership score. The dotted line shows the regression line.

A significant negative effect of the leadership score on D–ND activation was identified in the right inferior parietal lobule (Table 2; Figure 1). This finding remained significant even after correcting for multiple traits. The plots of D–ND activation against leadership score (Figure 1) implied a low neural response to mortality salience in individuals with a high leadership score. At this activation peak, the trait effects of the other Power to Live factors or self-esteem score on D–ND activation were not significant, even at the voxel-level statistical threshold (i.e., without correcting for searching over the entire brain; voxel-level *p* < 0.05/18; Table 3).

**TABLE 2:** Significant negative effect of the leadership score.

Structure	L/R	MNI coordinate				Cluster	
		*x*	*y*	*z*	*t*	# voxel	*p*
Inferior parietal lobule	R	46	−62	38	5.26	742	<0.001*
		50	−52	48	5.17		

Anatomical label, Montreal Neurological Institute (MNI) coordinate, t value of the activation peak and cluster size in the number of voxels (2 × 2 × 2 mm^3^), and its *p*-value are given for the significant negative trait effect. *p < 0.05/18 (Bonferroni correction).

**TABLE 3:** Effects of all the trait scores in the right inferior parietal lobule.

Trait	*t*	*p*
Leadership	−5.26	<0.001*
Problem solving	−2.53	0.008
Altruism	−1.79	0.042
Stubbornness	−0.33	0.374
Etiquette	−1.80	0.041
Emotion regulation	−2.64	0.006
Self-transcendence	−0.67	0.254
Active wellbeing	−2.06	0.024
Self-esteem	−2.29	0.015

The *t* values and associated p-values are given for the effects of all trait scores at the activation peak of the negative effect of the leadership trait in the right inferior parietal lobule (46, −62, 38). A negative *t* value indicates a negative effect. *p < 0.05/18 (Bonferroni correction).

No significant effects of the other Power to Live factors or self-esteem score were detected on D–ND activation in the entire-brain voxel-wise multiple regression analyses.

## 4. Discussion

A significant trait effect on neural response to mortality salience was identified for the leadership factor only, suggesting that the association of a resilient personality with the neural response to mortality salience may be better explained by a narrowly, rather than broadly, defined trait concept related to social coalition. Although the leadership score was significantly correlated with the self-esteem score, the latter did not significantly affect the neural response. Considering the conceptual similarity between the leadership factor of the Power to Live questionnaire and the secure attachment style, our findings may be in line with the view that the secure attachment style provides a key coping ability for mortality threat (Mikulincer and Florian, 2000; Taubman-Ben-Ari et al., 2002).

The negative effect of the trait on the inferior parietal lobule activation may be related to an immediate suppressive process previously suggested for the secure attachment style. In the TMT framework, two stages are assumed for the coping process after the presentation of a death reminder: an immediate suppression of accessibility to death-related thoughts (Hayes et al., 2010) and a worldview defense taking place as a prolonged unconscious process (Burke et al., 2010). A unique finding for the secure attachment style is its association with the former immediate process (Mikulincer and Florian, 2000). The remaining resilience-related personality traits are mainly related only to the latter process (Florian et al., 2001; Joireman and Duell, 2007; Hayes et al., 2010). A previous neuroimaging study demonstrated that the degree of activation in the frontoparietal cortices, including the right inferior parietal lobule, during the presentation of death-related words is associated with the death-relevance ratings of the presented death-related words (Shi and Han, 2013). Therefore, the identified negative effect of the leadership factor on the activation of this cortical region may be related to the presumed association between the secure attachment style and reduced death-thought accessibility (Mikulincer and Florian, 2000).

If our fMRI data primarily reflect an immediate suppressive process of accessibility to death-related thoughts, the observed lack of neural correlation with other factors may be reasonable. Hardiness and self-transcendence are associated with a reduced degree of worldview defense, but not with differences in death-thought accessibility (Florian et al., 2001; Joireman and Duell, 2007). If our fMRI analysis is sensitive only to the immediate response to death-related words, individual differences in prolonged processes, such as worldview defense, might not be detected (Burke et al., 2010). This is also supported by our finding of no significant effects of self-esteem, which primarily affects worldview defense, on neural activation (Hayes et al., 2010). This consideration may explain the inconsistent findings related to the effects of self-esteem on the immediate neural response to mortality salience in previous studies (Klackl et al., 2014; Yanagisawa et al., 2016; Luo et al., 2017).

Our findings highlight the unique functional characteristics of the immediate suppressive process in contrast to the prolonged worldview defense process, which has been discussed primarily in terms of the difference in time sequence. The current findings suggest that the former is affected by the narrowly defined social-coalitional tendency; that is, a leadership factor or the secure attachment style, mediated by the suppressed response in the right inferior parietal lobule. On the other hand, the latter has been implicated in the broadly-defined resilience-related personality trait (i.e., hardiness; Florian et al., 2001) and the broadly conceptualized anxiety-buffering resource (i.e., self-esteem; Schmeichel et al., 2009), although its neural correlates remain unclear. These findings seem congruent with a cautious stance regarding the existence of the worldview defense, as recent pre-registered studies have failed to replicate the foundational studies that the theory is based on (Schindler et al., 2021).

Our research supported the notion that the coping processes for mortality threat differ from those for general emotional distress, and the findings have some clinical implications. The resilient-personality trait and neural processes in response to the mortality threat identified in our study diverged from those previously linked to general emotional stress. In a previous fMRI study, the neural response to negative emotional pictures was negatively correlated with emotion regulation score in various prefrontal, sensorimotor, and temporal cortices (Sugiura et al., 2023). This finding is consistent with the impaired emotion regulation and dysregulated multilevel neurophysiological responses commonly implicated in mental disorders (Tanaka et al., 2022; Battaglia et al., 2023). Conversely, in our study, leadership was associated with a reduced parietal response during the mortality salience manipulation. This finding may be related to distinct aspects of psychopathology in mental disorders, where sociopsychological characteristics and observed response tendencies can be linked to mortality salience. That is, reduced proneness to social coalition (Zhang et al., 2022) and insecure attachment style (Mikulincer and Shaver, 2012) in mental disorders seem to be associated with diminished leadership and unsuppressed access to death-related thoughts, which may explain the increased anxiety-related behavior (Menzies et al., 2021) and worldview defense (Simon et al., 1998) seen in these patients.

## 5. Conclusion

Out of the eight factors of the Power to Live and self-esteem, a significant effect of the trait score on the neural response to mortality salience was observed only for the leadership factor. In individuals with a high leadership score, the neural response to mortality salience was low in the right inferior parietal lobule. Given the conceptual similarity between leadership and the secure attachment style, our findings may reflect an immediate suppressive process of death-thought accessibility, previously suggested for the secure attachment style.

Within the TMT framework, the findings highlight the unique characteristics of the immediate stage of the coping process with mortality salience, which may be affected by the narrowly defined social-coalitional trait. This stands in contrast with prolonged worldview defense, which may be affected by the broadly defined resilience-related personality traits. Our results lay the groundwork for future studies investigating the psychological processes underlying human social-coalition formation in the context of stress coping.

From a wider translational perspective, the findings may enhance understanding of the role of individual differences in the psychological response to mortality threat in psychopathology. Although the identified resilient-personality traits and neural processes arising in response to mortality threat are distinct from those related to general emotion regulation, dysfunction of the relevant coping process may party explain psychopathology through the association between a low level of social coalition and unsuppressed access to death-related thoughts.

## 6. Limitations and future directions

The current study, though insightful, is not without limitations. Four issues in particular merit further exploration to improve understanding of the psychological processes underlying human social-coalition formation in the context of stress coping.

First, the conceptual distinction and overlap between the two social-coalition-related traits should be examined. On the one hand, the attachment style typically addresses two-person relationships with a familiar other in an emotional context (Hazan and Shaver, 1987), whereas the leadership factor of the Power to Live questionnaire encompasses multi-party relationships in a utilitarian community context (Sugiura et al., 2015). On the other hand, early developmental processes are underscored for both traits (Bowlby, 1973; Matsuzaki et al., 2022). Second, specificity of the processes to mortality salience needs further investigation. Studies of the TMT framework have addressed processes specific to mortality salience using the negative emotional condition as control, similar to the present study. However, the social coalitional response may also occur in aversive situations unrelated to death, which may be better explained from an evolutionary perspective (Navarrete et al., 2004). Third, the distinct roles and consequences of the two stages need further exploration. Although the social-coalitional trait may contribute to the immediate suppression of death-thought accessibility, it may also facilitate certain aspects of worldview defense, such as those related to religiosity (Shults et al., 2018). Lastly, the non-significant effect of traits other than leadership should be carefully reassessed. The current findings are based on a relatively small sample (*n* = 34) of elderly individuals, who exhibit lower levels of worldview defense, despite maintaining the same level of death-thought accessibility as young people (Maxfield et al., 2007).

## Data availability statement

The data analyzed in this study is subject to the following licenses/restrictions: not publicly available due to the risk of identifying the participants from the reconstructed images. Requests to access these datasets should be directed to MS, sugiura@tohoku.ac.jp.

## Ethics statement

The studies involving human participants were reviewed and approved by the Institutional Review Board of the Graduate School of Medicine of Tohoku University. The patients/participants provided their written informed consent to participate in this study.

## Author contributions

KH and MS contributed to the study conception and design, performed the statistical analysis, and wrote the first draft of the manuscript. KH, KO, and TS prepared the experimental stimuli and conducted the MRI experiment. All authors contributed to the manuscript revision and read and approved the submitted version.
